# Quasi-guided modes resulting from the band folding effect in a photonic crystal slab for enhanced interactions of matters with free-space radiations

**DOI:** 10.3762/bjnano.14.27

**Published:** 2023-03-06

**Authors:** Kaili Sun, Yangjian Cai, Uriel Levy, Zhanghua Han

**Affiliations:** 1 Shandong Provincial Key Laboratory of Optics and Photonic Devices, Center of Light Manipulation and Applications, School of Physics and Electronics, Shandong Normal University, Jinan 250358, China; 2 Department of Applied Physics, The Hebrew University of Jerusalem, Jerusalem, Israel

**Keywords:** guided modes, light–matter interactions, photonic crystal slab

## Abstract

We elucidate that guided modes supported by a regular photonic crystal slab structure composed of a square lattice of air holes in a silicon slab will transition into quasi-guided (leaky) modes when the radius of every second column of air holes is changed slightly. This intentional geometric perturbation will lead to a doubling of the period in one direction and the corresponding shrinkage of the first Brillouin zone. Because of the translational symmetry in the *k*-space, leaky waves inheriting the spatial dispersion of the original guided modes, which do not interact with external radiation, will appear with the dispersion curves above the light cone. Our results show that ultrahigh Q-factor resonances with large operating bandwidth can be achieved. Interestingly, the perturbation in only one direction of the photonic lattice will lead to an in-plane wave number-dependent resonance characteristic in both directions. Our numerical results demonstrate a local enhancement of the electric field magnitude by the order of 10^2^, which is even more significant than those in most plasmonic structures. These quasi-guided modes with superior properties will provide a new platform for efficient light–matter interactions.

## Introduction

Photonic resonances with the possibility of free-space excitation (i.e., leaky modes) and large local electromagnetic field enhancement are central for the manipulation of light–matter interactions. Optical resonators of various forms have been exploited for this purpose. What follows are a few representative examples investigated in the last several decades: Photonic crystal cavities are realized when small disorders or defects are introduced into large-scale periodic structures [1]. Extremely high Q-factors can be achieved thanks to the bandgap associated with the periodic structure, which prevents the leakage of radiation into the surrounding environment. Whispering gallery modes supported by dielectric spheres or suspended disks made of high-index materials are another example of resonances to provide ultrahigh Q-factors [2]. However, above structures are still bulky. For example, the photonic crystal cavities need the surrounding periods to provide the bandgap, which is not favorable for nanoscale applications. Plasmonic nanoantennas [3], although with relatively low Q-factors resulting from material dissipation, still provide a large level of field enhancement due to the deep-subwavelength level of mode confinement. As new alternatives to plasmonic nanostructures, all-dielectric nanostructures supporting Mie resonances [4] and quasi-bound state in the continuum (QBIC) modes [5] have attracted significant attention in nanophotonic research, with the latter proposed to address the problem of radiation losses associated with the former. A large variety of novel applications benefiting from such optical resonances have been demonstrated in all aspects of light–matter interactions, ranging from optical generation [6], propagation [7], nonlinear processes [8] to signal detection [9] and collection, to name a few. Although QBIC resonances in all-dielectric nanostructures have become a popular and mainstream approach to enhance light–matter interactions, as derivatives of ideal BIC resonances, which are associated with isolated or discrete points of high symmetry in the ω–*k* space [10], they still suffer from very limited operating bandwidth. As a result, the QBIC resonances are not suitable for many important optical applications where multiple or spectrally tunable inputs are required simultaneously. Consequently, new mechanisms are still explored to realize novel photonic components with additional advantages besides a high Q-factor. These are, for example, phase gradient metasurfaces and spatial beam splitters [11], metasurfaces that produce narrow-band spatially tailored wave fronts [12], and zigzag arrays of dielectric disks with ultranarrow bandwidth resonances over a large spectral band [13]. Some new attempts to engineer the radiation environment to achieve so-called lines of BICs have emerged quite recently [14]. But the idea and reported results require very complicated geometries [15], which are challenging to fabricate.

## Results and Discussion

In this work, we propose a fundamentally different approach to realize optical leaky resonances that can combine all the advantages of the above resonances, that is, ultrahigh Q-factors, huge local electric enhancement, and intermediate mode volume, while providing a large operation bandwidth. Unlike QBIC resonances, we start from guided modes (GMs) whose optical fields are well confined within the geometry and have no access to external radiation. The GMs have typical continuous and one-dimensional dispersion curves below the light line over a large bandwidth. This leaky resonance is generated through band folding, which occurs when a perturbation is introduced into a regular periodic structure to have its period increased and the first Brillouin zone (FBZ) shrunk. Because of the translational symmetry in the *k* space, the GMs with infinite Q-factors supported by the original lattice will appear as new leaky resonances with the dispersion curves above the light cone in the new structure. These resonances, termed quasi-guided modes (QGMs), will inherit the spatial dispersion of the original GMs, with Q-factors significantly dependent on the level of perturbation. As a result, they feature ultrahigh Q-factors while the resonance can be tuned by the lateral wave vector. The QGMs outperform QBIC resonances, which can only operate within a narrow bandwidth, even at a wave number largely different from that of the original BIC resonance.

We should note that a similar band-folding effect has been proposed in the literature to improve the angular tolerance in the reflection of resonant grating filters with doubly periodic structures [16–17]. Other structures, such as diatomic [18] or dimerized [19–20] gratings, have been also investigated in recent years, but mainly with emphasis on the far-field spectrum, using one-dimensional (1D) grating structures. In addition, band folding was also employed to realize terahertz radiation from difference frequency generation (DFG) by using 1D leaky modes of binary waveguide gratings [21] and to manipulate the radiation coupling in the vertical directions in some photonic crystal cavities [22–23]. A similar structure of a ZnO photonic crystal slab (PCS) with doubled periods in both directions has been proposed to realize low-threshold polariton lasers [24]. However, we show in this work that, even when the period increase and the accompanied FBZ shrinking occurs only along one direction of the two-dimensional periodicity, the resonance still depends on the in-plane wave vector along both directions. This suggests the possibility of resonance tuning over an extended bandwidth by using the incident angle along two different directions as the tuning mechanism. More importantly, we further illustrate that these QGM resonances have a significantly enhanced local electric field, which is even larger than that of most plasmonic nanoantennas, suggesting the great potential of these QGMs for enhanced light–matter interactions.

**Figure 1:**
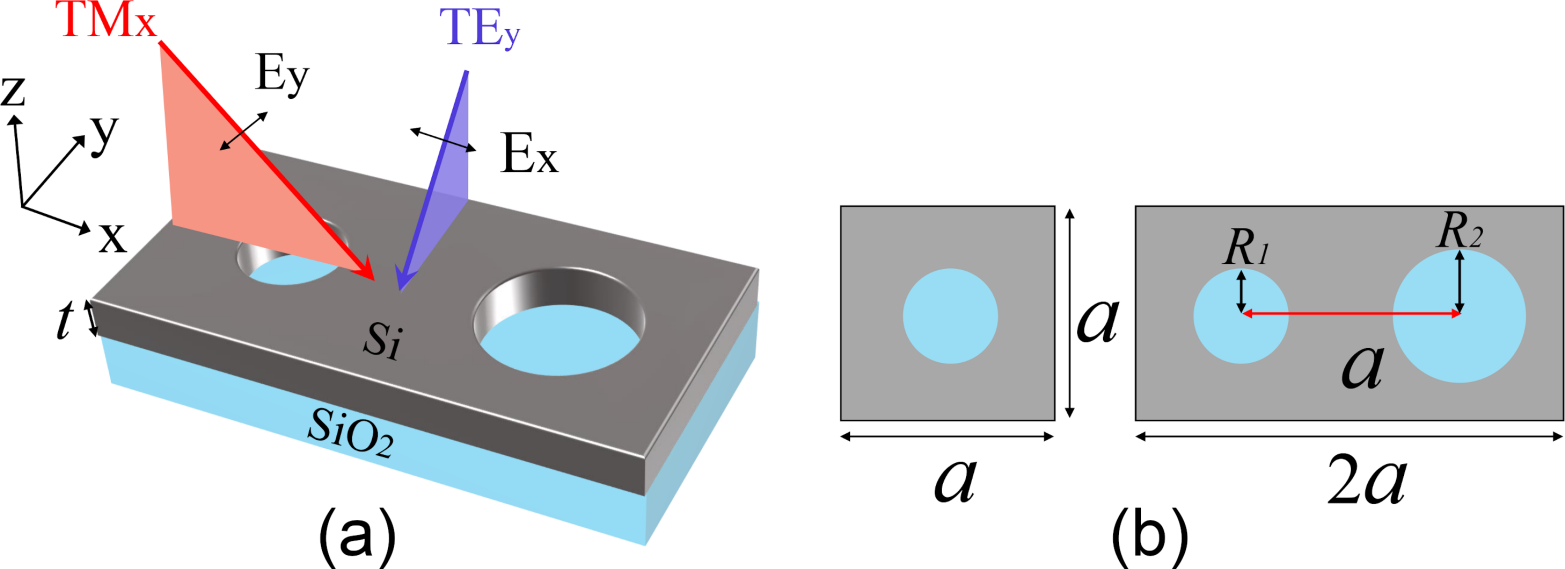
(a) Schematic of the photonic crystal slab structure of air holes in a silicon layer. (b) Top view of the unit cells for two cases. Left: the square lattice of the original structure, right: the distorted rectangular lattice with a period of 2*a* × *a*.

**Figure 2:**
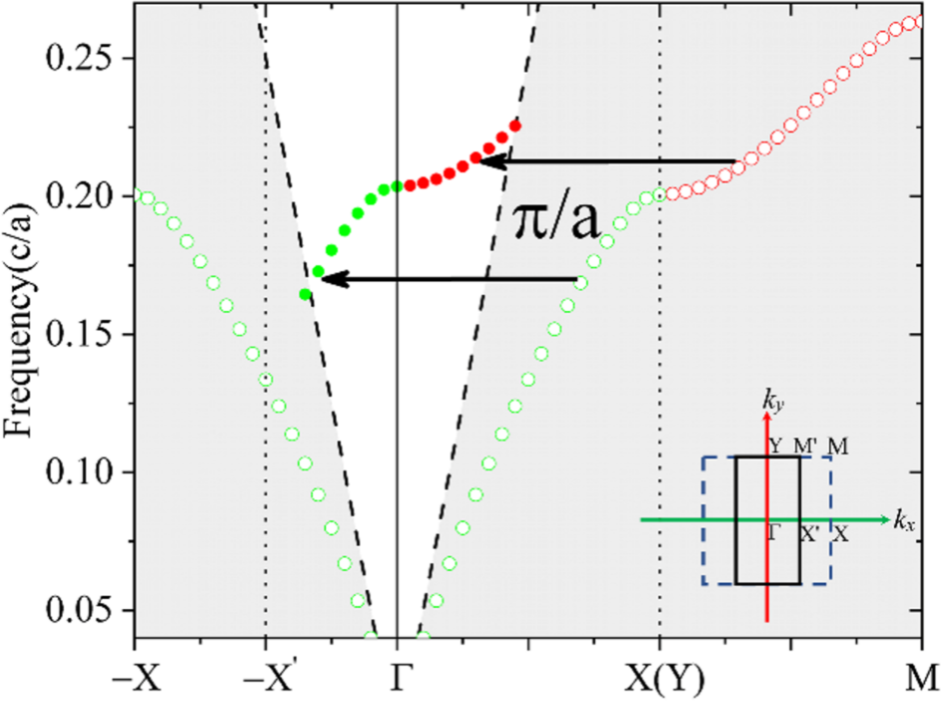
Dispersion curves of the GMs (hollow circles) supported by the PCS of air holes in SOI and the QGMs (solid circles) supported the distorted lattice where the radius of every second column of air holes is changed to *R*_2_. The inset shows the FBZ for the two cases. The green and red colors denote the dispersion along the *k_x_* and *k_y_* direction, respectively.

We use GMs supported by a regular PCS structure composed of a square lattice of air holes perforating a thin silicon (refractive index: 3.45) film on a silica (refractive index 1.45) substrate as an example to demonstrate that these modes can be switched to QGMs with ultrahigh Q-factors over a large operating bandwidth, as shown in Figure 1. When all air holes have the same radius, the whole structure represents a two-dimensional PCS structure with a square primitive unit cell. With the period *P_x_* = *P_y_* = *a* along both *x* and *y* directions, this structure is known to support a set of well-confined GMs with no external radiation [25], which lay out the foundation for integrated photonic elements in the PCS. The lines of empty circles in Figure 2 present the dispersion curve for the GMs along ΓΧ and XΜ directions in the FBZ supported by the square lattice with *a* = 400 nm, *R*_1_ = 100 nm, and *t* = 220 nm. The results were obtained by using the eigenfrequency analysis and lateral Fouquet boundary conditions implemented in the commercial finite-element method software Comsol Multiphysics. All numerical models are built with 3D structures. The size of the tetrahedral mesh was tested to ensure the numerical convergence of the calculated results. It is seen that the dispersion curve of the GMs *f*_0_(*k_x_, k_y_*) is well below that of the light cone (this region is displayed with a dark background). When the radius of the air holes in every second column is increased by a quantity of δ to *R*_2_ = 120 nm, the period along the *x* direction will be doubled to be *P*_2_*_x_* = 2*a* while it remains unchanged in the *y* direction. As a result, the FBZ shrinks in the *x* direction and its shape changes from a square to a rectangle, as shown in the inset of Figure 2. With the period in the *k_x_* direction halved to 2π/*P*_2_*_x_* = π/*a*, one has the dispersion equation *f*(*k_x_, k_y_*) in the distorted lattice as:


[1] $$f\left(k_{x},k_{y}\right)=f\left(k_{x}-\pi /a,k_{y}\right).$$

When the perturbation introduced into the lattice is weak, the distorted lattice remains approximately the same as the undistorted, and so are the supported resonance frequencies. Then we have


[2] $$f\left(k_{x}-\pi /a,k_{y}\right)\approx f_{0}\left(k_{x}-\pi /a,k_{y}\right).$$

Combining Equation 1 and Equation 2, we obtain the following equation:


[3] $$f\left(k_{x},k_{y}\right)\approx f_{0}\left(k_{x}-\pi /a,k_{y}\right),$$

which suggests that dispersion curves with similar profiles as the GMs in the PCS around the Χ point will appear around the Γ point in the distorted lattice. In other words, the dispersion curve of GMs along ΓX and XM directions in the original square unit cell will be translated to the −X'Γ and ΓΥ directions in the new lattice, respectively. Since the wave numbers close to Γ point are relatively small, the majority of the translated dispersion curves will be located above the light cone in the distorted lattice, suggesting leaky resonances.

The curve composed of solid circles in Figure 2 presents the calculated dispersion for the QGMs of the rectangular primitive cell shown on the right side of Figure 1b, where *R*_2_ is set to be 120 nm. The eigenfrequencies are found around the same band as the GMs. The results are entirely consistent with the above predictions from Equation 3. It is quite clear that the dispersion curves of the QGMs around the Γ point have roughly the same profiles as the original GMs around the Χ point, both in the *k_x_* and *k_y_* directions. As a result, although the period remains unchanged along the *y* direction (*P_y_* is still *a*), the dispersion along *k_y_* is also located above the light cone and resembles the profile of *f*_0_(*k_x_, k_y_*) along the XΜ direction. A weaker spatial dispersion of the QGMs is present in the *k_y_* direction compared to the *k_x_* direction, which is the same as for the GMs.

The total Q-factor (*Q*_total_) of a resonance observed in the far-field spectrum is determined by the Q-factors of radiation (*Q*_rad_) and absorption (*Q*_abs_) [26]:


[4] $$\frac{1}{Q_{\text{total}}}=\frac{1}{Q_{\text{rad}}}+\frac{1}{Q_{\text{abs}}}.$$

**Figure 3:**
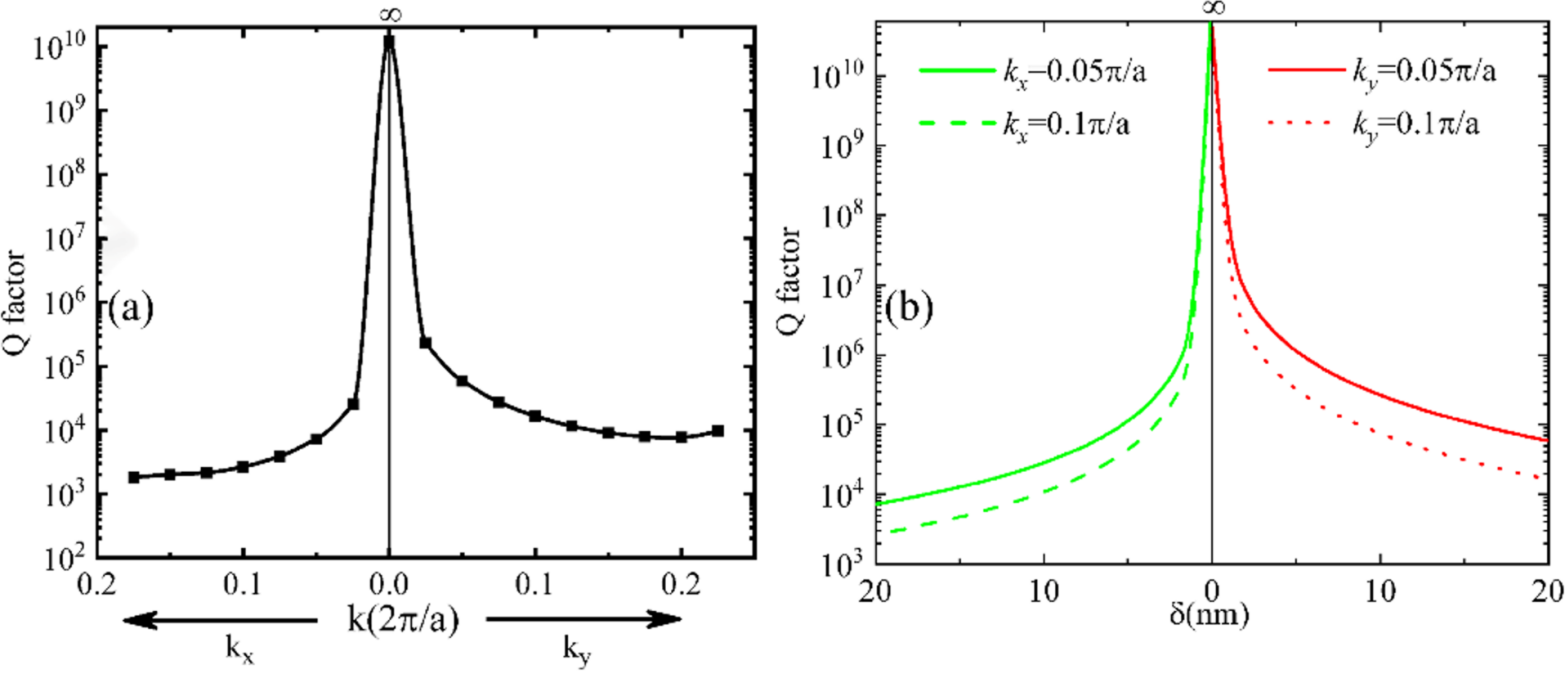
(a) Q-factors along the dispersion curves of QGMs in Figure 2. (b) Q-factor as a function of the level of perturbation at two points along two directions.

Because the absorption loss of materials is not considered here, that is, *Q*_abs_ is infinite, *Q*_total_ is determined solely by *Q*_rad_ of the structure. Its value or the radiation loss can be obtained from the real and imaginary parts of its complex eigenfrequency from the numerical calculations. The calculated Q-factors of these QGMs are presented in Figure 3a. The value is infinite at the Γ point, which arises from an ideal BIC resonance of the symmetry-protected type. This is because the new periodic structure, even with the radius of the air holes in every second column changed, still exhibits a mirror symmetry across the central *xz* and *yz* planes of all air holes. Further away from the Γ point, the Q-factor decreases for larger wave numbers but maintains overall large values (above 10^3^) for all resonances. To have a moderate level of Q-factors (i.e., measurable in practical experiments) for the transmission spectra presented in the subsequent part, we used intentionally a stronger perturbation with a δ value of 20 nm. We note that the overall Q-factors will be significantly increased if a weaker perturbation is introduced. The dispersion of the GMs is located well below the light line, preventing any outward radiation due to total internal reflection. In other words, the Q-factors of all GMs are infinite since we ignore the material absorption in the lossless dielectrics. When the period-doubling perturbation is applied and the new QGMs are formed because of the folding of the FBZ, the coupling efficiency between free-space radiation and the QGMs is still very low, leading to the occurrence of high Q-factor resonances. Intuitively, the Q-factors highly depend on the level of perturbation. In addition, since the spatial dispersion of the original GMs is retained in the QGMs, one can have ultrahigh Q-factors over a large bandwidth, and the resonance can be tuned by changing the wave vector or, equivalently, the incident angle of external excitations. This is in huge contrast to QBIC resonances, whose frequency is limited within a narrow band close to the frequency of the original BIC resonance from which the QBIC resonances are derived. Similar to QBIC resonances, the Q-factors exhibit a strong dependence on the level of perturbation and increase significantly as the perturbation decreases. The value of the Q-factor will approach infinity as δ approaches zero, where the lattice returns to the regular square lattice of air holes (*P_x_* decreases from 2*a* to *a*) and the QGMs switch back to GMs. Interestingly, the trend of *Q* approaching infinity when the perturbation vanishes is true for any resonance along the QGM dispersion curve. Figure 3b presents the calculated Q-factors at two randomly selected points along *k_x_* and *k_y_* for the QGMs and the dependence of *Q* on the extent of the perturbation is clearly seen. We should note that this behavior is another feature of the QGMs significantly different from those of QBIC resonances. The operating bandwidth of QBIC resonances significantly depends on the level of perturbation introduced into the geometry to transform the BIC resonance into QBICs. The bandwidth is smaller if the perturbation is weaker, which is the case when one aspires for a high Q-factor. As the geometrical perturbation decreases, the dispersion curve of the QBIC resonances will shrink to a single point in the ω–*k* space, which represents the BIC resonance. For the QGMs, the operating bandwidth is not affected at all by the level of perturbation. Instead, it is determined by the spatial dispersion of the GMs in the PCS. Thus, the ultrahigh Q-factors can be maintained over the same broad bandwidth, regardless of the level of perturbation. All these properties of the QGMs make it possible to realize superior leaky modes with ultrahigh Q-factors and a value of *Q* completely controlled by the extent of perturbation over the same bandwidth. Compared to the 1D periodic structure, the operation bandwidth of a 2D structure extends by exploiting the changes of the wave number in a direction different from the direction of lattice change.

**Figure 4:**
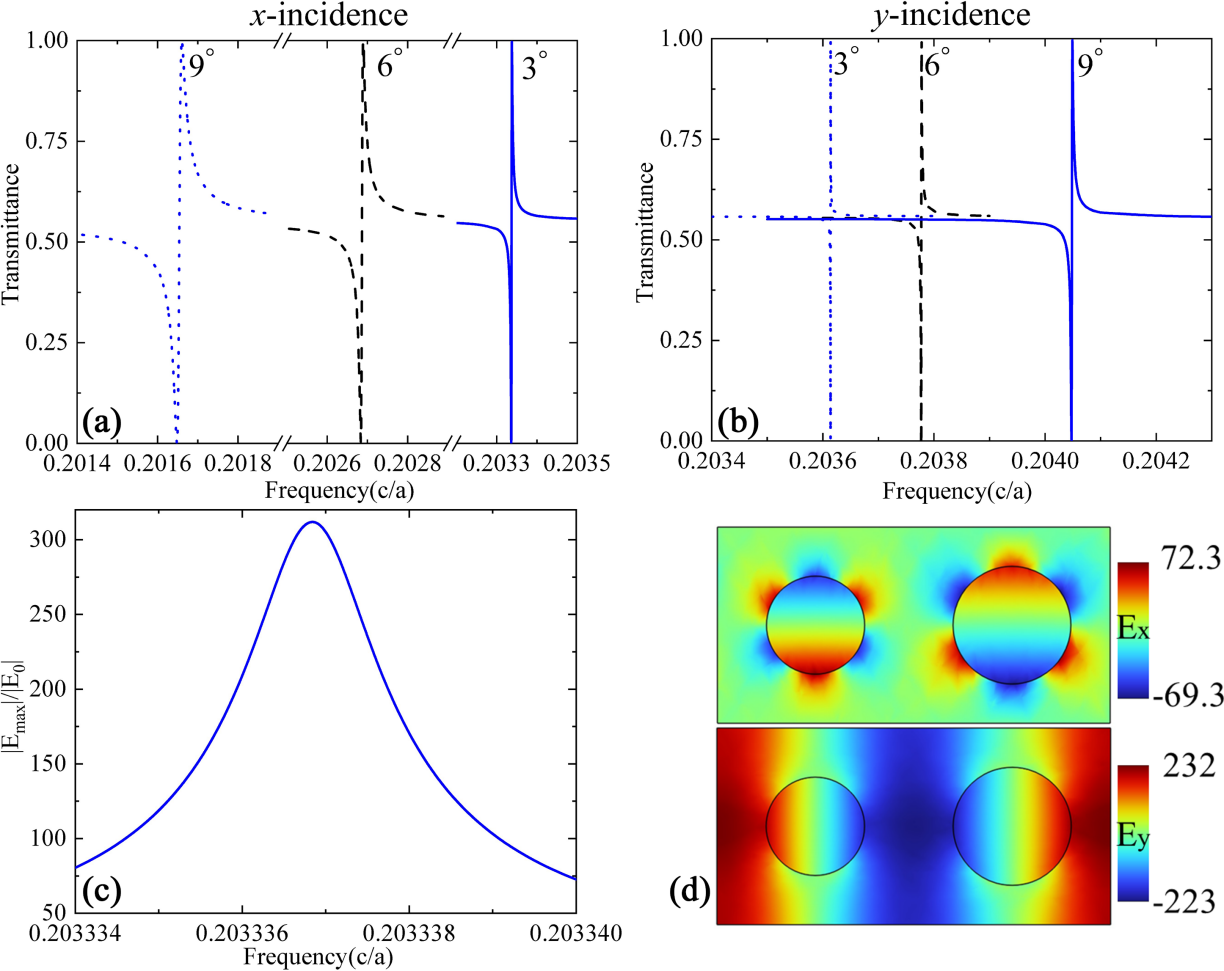
Transmission spectra at different incident angles with the incidence in (a) the *xz* plane and (b) the *yz* plane. (c) The maximum electric field magnitude normalized to that of the incident wave across the central plane of the Si layer. (d) Typical distributions of *E_x_* and *E_y_* along the central plane of the silicon layer.

To have a straightforward demonstration of tuning the resonance via the incident angle, we present in Figure 4 the calculated transmission spectra for three different incident angles of 3°, 6°, and 9° along both *x* and *y* directions. We note that when the period-doubling perturbation is absent (*R*_1_ = *R*_2_), the structure returns to a regular PCS, which supports the well-known guided mode resonances in a frequency-doubled spectrum range [27]. The guided mode resonances have relatively broad bandwidths compared to QGM resonances, and their properties have been well documented in the literature. In the spectrum of our interest, the regular PCS only supports broadband Fabry–Pérot resonances, and the transmission exhibits no sharp features, because only well-confined GMs are supported. However, when the perturbation is applied, QGMs will be formed and new sharp resonances will be superimposed onto the transmission spectrum. The setup of the incident beams with respect to the structure can be found in Figure 1a, where the electric field of *E_y_* is used to excite the QGMs. A redshift of the resonance for a larger incident angle is observed for TM*_x_* along the *x* direction, while the trend is opposite for TE*_y_* along the *y* direction. Judging from the bandwidth of the resonance, one can see that the Q-factor decreases slightly at a larger incident angles for the incidence along the *x* direction, while it increases for the incidence along the *y* direction. All these results are consistent with the dispersion curves in Figure 2 and the evolution of Q-factors as a function of the wave number in Figure 3a. Figure 4d presents typical distributions of the real part of both *E_x_* and *E_y_* at the resonance frequency across the central plane of the silicon layer under an incidence angle of 3° along the *x* direction. It is seen that *E_x_* exhibits a symmetric profile while *E_y_* has the opposite distribution within individual holes along the *x* direction, where the lattice distortion happens. The distribution of the electric field mainly within the air holes is due to symmetry reasons. For our structure, in which the radius of every second column of holes is changed, mirror symmetry is still retained across the center of each hole the along *x* direction, which ensures an effective coupling between the modes with *x*-polarized plane waves. This kind of field distribution in Figure 4d is useful for sensing applications. For other applications where one would like to have the main field within the dielectrics, another kind of perturbation by moving the position of every second column of holes could be used instead. The distributions in Figure 4d confirm that the QGMs around the Γ point inherit the same mode profiles of the original guided mode at the boundary of the FBZ (the Χ point), where the field distributions are anti-symmetric in the ΓΧ direction. In addition, it is known that the maximum local field enhancement is determined by the resonance Q-factor and the mode volume [26]. For periodic structures, a discussion of the mode volume calculation can be found in [28]. The intermediate mode confinement within the photonic crystal slab structure and the ultrahigh Q-factors of the QGMs make it possible to obtain a huge electric field enhancement. Figure 4c presents the maximum local electric field magnitude normalized to that of the incident plane wave. It can be seen that an enhancement factor of 312 can be achieved for an incident angle of 3° along the *x* axis. This number decreases to 156 and 107 for incident angles of 6° and 9° along the *x* axis, respectively, and increases to 950, 477, and 319, respectively, for incident angles of 3°, 6°, and 9° along the *y* axis. The trend of the level of enhancement is consistent with that of the Q-factor as a function of the incident angle shown in Figure 3. We should note here that all these values of local electric field enhancement are generally higher than those that can be achieved with a regular guided resonance in a similar PCS structure supporting Fano-type resonances [27] or with most plasmonic nanoantennas [3]. This is because of the high value of *Q* and the relatively low mode volume of these QGMs [26]. We further note that the maximum electric field enhancement is located within the air holes (from the magnitude of the electric field, which is not shown in Figure 4), where the mirror symmetry results in a large spatial overlap of the mode with the electric field of the incident plane wave [19–20]. If another kind of period-doubling perturbation is used, for example, by shifting the position of every second column of air holes, the mirror symmetry will be maintained within the dielectric material between the air holes, where the local electric field enhancement will occur.

## Conclusion

We have presented in this work the superior properties of the QGMs that occur when a perturbation is introduced in a regular PCS. The QGMs inherit the spatial dispersion of the GMs supported by the PCS and consequently feature ultrahigh Q-factors, which can be controlled by the level of perturbation over a large bandwidth. The huge local field enhancement, even higher than that in plasmonic nanoantennas, has been demonstrated using numerical simulations. Although a PCS structure in the form of air holes in a silicon slab is used for demonstration, we note the same physics can be extended to other periodic structures such as arrays of silicon rods. The huge local field enhancement together with the possibility of resonance tuning by the incident angle over a large bandwidth make the QGMs a competitive platform for enhanced light–matter interactions and novel applications. For example, in some nonlinear applications, the interactions between the incident light and the medium need to be enhanced simultaneously at multiple wavelengths with largely different values. This requirement can easily go beyond the capability of QBIC resonances. However, it can be easily fulfilled using QGMs by simply choosing the proper incident angles.
